# Elevated Heme Oxygenase-1 Correlates With Increased Brain Iron Deposition Measured by Quantitative Susceptibility Mapping and Decreased Hemoglobin in Patients With Parkinson’s Disease

**DOI:** 10.3389/fnagi.2021.656626

**Published:** 2021-03-18

**Authors:** Jinghui Xu, Chi Xiao, Weizheng Song, Xiangqin Cui, Mengqiu Pan, Qun Wang, Yanqiu Feng, Yunqi Xu

**Affiliations:** ^1^Department of Neurology, Nanfang Hospital, Southern Medical University, Guangzhou, China; ^2^Department of Rehabilitation Medicine, The Third Affiliated Hospital, Sun Yat-sen University, Guangzhou, China; ^3^Department of Neurosurgery, the Eighth People’s Hospital of Chengdu, Chengdu, China; ^4^Department of Neurology, Guangdong 999 Brain Hospital, Guangzhou, China; ^5^Guangdong Provincial Key Laboratory of Medical Image Processing, Southern Medical University, Guangzhou, China

**Keywords:** Parkinson’s disease, heme oxygenase-1, brain iron deposition, low hemoglobin, quantitative susceptibility mapping

## Abstract

**Background**: Brain iron deposition, low hemoglobin (HGB), and increased heme oxygenase-1 (HO-1) have been implicated in Parkinson’s disease (PD). However, the association among them in PD is poorly studied.

**Objective**: To explore the association of the level of HO-1 with brain iron deposition and low level of HGB in PD.

**Methods**: A total of 32 patients with PD and 26 controls were recruited for this study. C57BL/6 male mice were used in generating 1-methyl-4-phenyl-1,2,3,6-tetrahydropyridine (MPTP)-induced chronic PD model. The Levels of serum HO-1 and HGB of human subjects and mice were assayed by ELISA, blood routine test, respectively. Quantitative susceptibility mapping (QSM) was used to quantitatively analyze brain iron deposition in human subjects and mice. HO-1 inhibitor (Sn-protoporphyrin, SnPP) was used to suppress the function and expression of HO-1 in PD mice. Correlations between the concentration of serum HO-1 and iron deposition of the region of interests (ROIs), levels of HGB, between the three factors mentioned above, and scores of clinical scales were explored in PD patients.

**Results**: This study revealed significant elevation of the serum HO-1 concentration, iron deposition within bilateral substantial nigra (SN), red nucleus (RN), and putamen (PUT) and decrease of HGB level in PD patients. There was a significantly positive correlation between the serum HO-1 concentration and iron deposition within SN, an inverse correlation between the serum HO-1 concentration and HGB level in PD patients. A significant increase in HO-1 expression of serum and iron deposition in SN was also observed in the PD mouse model, and the SnPP could significantly reduce iron deposition in the SN.

**Conclusions**: The high level of HO-1 may be the common mechanism of iron deposition and low HGB in PD. Therefore, the findings presented in this study indicate that HO-1 correlates with brain iron deposition and anemia in PD.

## Introduction

Post-mortem and *in vivo* studies have demonstrated that high iron content is a prominent pathophysiological feature of PD (Dexter et al., 1987; Berg and Hochstrasser, 2006; Jin et al., 2011). Excessive iron induces cell death by altering the state between ferrous and ferric forms (Barnham et al., 2004). A series of studies have demonstrated the impact of iron deposition on the pathological aggregation of alpha synuclein (Ostrerova-Golts et al., 2000; Li et al., 2010; He et al., 2015). Although iron deposition is an important pathological hallmark of PD and promotes its pathological progress, the root cause of brain iron deposition is still unclear (He et al., 2015). Many theoretical hypotheses have been used to explain iron deposition in the nigrostriatal system, including an increase of iron intake, change of iron transporter, and increased permeability of the blood-brain barrier (Ward et al., 2014). Presently, no theory explains total iron deposition, thus the need for in-depth studies.

The change of HGB in patients with PD has been attracting the attention of researchers. Several studies revealed that patients with lower HGB were more susceptible to PD (Savica et al., 2009). Our previous study (Deng et al., 2017) showed that, compared with non PD patients, the HGB in PD patients decreased significantly, to the level of anemia, though a small sample-size study exhibited that the levels of HGB did not change in PD patients (Madenci et al., 2012). It is generally believed that HGB decreases significantly in PD patients during the whole period of the disease. However, the root cause of decreased HGB as well as the underlying relationship between PD and decreased HGB is not clear.

To date, most studies have revealed decreased HGB and iron deposition, but how they interact with each other in PD is still unknown. Considering that iron in the human body mainly exists in HGB, it is speculated that there is an internal correlation between iron deposition and reduction of HGB.

Heme oxygenase 1 (HO-1) is an enzyme that catalyzes the degradation of heme into biliverdin or bilirubin, ferrous iron, and carbon monoxide. The expression of HO-1 is maintained at a low level and is restricted to some scattered neurons and glia in the central nervous system (Barañano and Snyder, 2001). However, after exposure to oxidative challenge, the expression of HO-1 protein significantly increases. Recently, some studies revealed increased HO-1 in astrocytes, dopaminergic neurons in the substantia nigra and in the serum of PD patients (Schipper, 2004; Mateo et al., 2010). The increased levels of HO-1 in PD might reflect an activated antioxidant reaction induced by chronic oxidative stress state in PD patients.

Considering the close association between HO-1 and heme catabolism as well as iron metabolism, it was hereby hypothesized that over-expression of HO-1 may influence brain iron deposition and HGB levels in patients with PD. To the best of our knowledge, the association between the level of HO-1, brain iron deposition, and lower HGB level in patients with PD has not been reported. It is against this background that, this study was conducted.

The imaging method for investigating brain iron deposition has been applied extensively. Particularly, the application of QSM in the detection of iron deposition in PD patients has already been reported in recent studies (He et al., 2015; Wei et al., 2019) due to its capacity for distinguishing iron-rich structures from the surrounding background (Li et al., 2011; Xuan et al., 2017). Currently, QSM has become an increasingly useful diagnostic tool for diseases associated with iron deposition (Eskreis-Winkler et al., 2017).

This study utilized QSM to determine iron deposition in various brain regions of patients with PD as well as in age- and gender-matched control subjects, and in the MPTP-induced PD mice model. This study aims to explore whether increased HO-1 levels are correlated with iron deposits in selected brain regions and lower HGB levels in PD. Investigating this relationship would explain the pathogenesis of iron deposition and lower HGB in PD and provide potential therapeutic targets and diagnostic markers.

## Materials and Methods

### Patients and Ethics Statement

This study was approved by the Ethics Committee of Nanfang Hospital. All subjects provided written informed consent before the start of the study. Thirty-two PD patients were enrolled from the Nanfang Hospital of Southern Medical University and Guangdong 999 Brain Hospital. PD diagnosis was conducted by at least two experienced neurologists according to the MDS clinical diagnostic criteria for PD (Postuma et al., 2015). Additionally, a total of 26 controls were recruited by at least two experienced neurologists who were blinded to the research objectives. Most of the patients in this group exhibited minor neurological deficits, such as mild headache, dizziness, etc. Both the PD patients and control groups (CG) were from Han ethnicity. All controls did not have extrapyramidal symptoms, brain trauma, hypothyroidism, psychiatric disease, anosmia, constipation, insomnia, and cognitive decline. Patients and controls who were malnourished, or had eating disorders, diagnosed with gastrointestinal disease or thalassemia, were not enrolled in this study.

### Human Nervous System Assessment and Neuropsychological Testing

The severity of PD was assessed by the MDS-Unified PD Rating Scale and the Hoehn & Yahr Scale (Goetz et al., 2008) while cognitive level was assessed by CMMSE (Cui et al., 2011). Also, Hamilton Depression Scale (HAMD scale) and Hamilton Anxiety Scale (HAMA scale) were used to assess the mental state of the participants; PD Sleep Scale was used to assess sleep quantity and non-motor symptom scale (NMSS) was used to assess some non-symptoms, such as hyposmia and constipation. The scale was evaluated by a trained and experienced specialist who was also blinded to the research objectives. The onset of PD was recorded as the first time when a patient experienced typical motor symptoms such as bradykinesia, resting tremor, and rigidity.

### Mouse Model

All experiments were approved by the Institutional Animal Care and Use Committee of Nanfang Hospital, Southern Medical University, and performed following the National Institutes of Health Guide for the Care and Use of Laboratory Animals. MPTP-HCl (Sigma, Italy) was dissolved in saline; 3-month-old male C57BL/6J mice (Laboratory Animal Center of Nanfang Hospital, China) were divided into four groups (*n* = 5 for each group). The control group received saline as a vehicle. MPTP (20 mg/kg i.p.) was injected twice a week for 5 weeks in the MPTP group (Jackson-Lewis and Przedborski, 2007), SnPP group received SnPP (40 μmol/kg/times) twice a week for 5 weeks and MPTP plus SnPP group received SnPP (40 μmol/kg/times) twice a week for 5 weeks an hour before MPTP injection. Twenty-four hours after the last MPTP injection, QSM examination and tissue sampling were performed.

### Preparation and Preservation of Blood Samples

Whole blood and blood for serum preparation were collected from PD patients after 8 h of fasting via median cubital vein and mice by cardiac puncture. The samples were then put into EDTA-coated tubes and drying tubes, respectively. For serum preparation, blood was clotted in drying tubes for 2 h at room temperature and centrifuged at 1,500 *g* at 4°C for 10 min and the serum was carefully collected for analysis of HO-1.

### HGB Testing

To observe the changes of HGB in PD patients and PD mice models, A KX21 automatic blood cell analyzer (Sysmex, Japan) and an Abaxis VetScan HM5 hematology analyzer (Abaxis, Union City, CA, USA) were used for routine blood tests from patients and mice, respectively.

### Enzyme-Linked Immunosorbent Assay (ELISA)

HO-1 Sandwich ELISA kits (ab207621, Massachusetts, USA and LS-F4086, LSBio, USA) were used to measure the level of HO-1 in serum obtained from the PD patients and mice, respectively according to the manufacturer’s instructions.

### QSM Image Acquisition for PD Patients

A total of 24 patients and 20 age-and gender-matched controls were subjected to QSM test via clinical 3T MR imaging system (MK750, GE Healthcare, Milwaukee, WI), and data were acquired. Imaging parameters were as follows: TE/TR = 21 ms/15 ms; matrix size = 183 × 156; slice thickness = 2 mm; FOV = 182 × 220 mm^2^; slice space = 0.6 mm; and Flip Angle = 10°. Before the reconstruction of QSM, two researchers independently graded the quality of the images and the unqualified images were excluded.

### Image Acquisition for PD Model

For animal research, five mice in each group with qualified QSM images were included for analysis of iron deposition in the brain. The specimens were scanned on a 7 Tesla system with a Bruker console (Bruker Biospin, PharmaScan70/16, US). To obtain magnetic susceptibility maps, the specimens were scanned with a multi-echo 3D gradient-echo sequence (eight echo) under the following imaging parameters: TE1/△TE/TR = 5.37 ms/8.07 ms/250 ms, flip angle 35°.

### QSM Image Reconstruction

QSM was processed using the STI Suite software (Duke University) after phase images in DICOM format were retrieved from the MRI scanner, as previously described (Kressler et al., 2010; Argyridis et al., 2014). Briefly, the phase images were unwrapped using a Laplacian-based phase unwrapping technique that relied on the sine and cosine functions of the phase angle (Li et al., 2011). The background phase was effectively removed using the harmonic artifact reduction for phase data with varying kernel sizes (VS.HARP; Li et al., 2011). The streaking artifact reduction for QSM (STAR-QSM; Kressler et al., 2010) was then used to improve the accuracy of magnetic susceptibility at the tissue edge. After the tissue field was inverted from field to source, the QSM images were finally obtained.

### Statistical Analyses

All continuous variables (the HO-1, HGB concentrations, the age, and clinical scale) were expressed as the means ± standard deviations while categorical variables were expressed as frequencies and percentages. The data for serum HO-1, HGB, and the QSM values of the ROIs were symmetrically distributed, and group mean values were compared using the independent-samples T-test or one-way ANOVA if the data had no variance(see the “Results” section for further details). A partial correlation coefficient was determined using age as a covariate to evaluate the relationship between the concentration of serum HO-1, levels of HGB, and the QSM values of the ROIs. Spearman correlation coefficient was determined to evaluate the relationships between the three factors mentioned above and scores of clinical scales, as the scores of clinical scales were abnormal distribution. A *p*-value of less than 0.05 was considered to be statistically significant. All data were analyzed using the standard statistical package SPSS v. 22.0 (IBM, Chicago, IL, USA), and graphical representations were made using GraphPad Prism v. 7 (GraphPad Software, Inc. La Jolla, CA, USA).

## Results

### Demographic and Clinical Characteristic

All recorded demographic and clinical characteristics of the subjects are presented in Table 1 and there were no significant differences in patient’s ages. All controls did not show typical non-motor symptoms for PD patients. Twenty-four PD cases and 20 controls with qualified QSM images were included for analysis of brain iron deposition (Table 1).

**Table 1:** Demographics of patients with Parkinson’s disease (PD) and control group (CG).

	PD	CG	*p*
*N*	32	26	
M/F, *n*	11/21	12/14	0.362^a^
Age, years	60.97 ± 1.48	56.19 ± 2.34	0.092^b^
Duration, years	4.60 ± 1.10	/	/
Hoehn-Yahr stages	2.46 ± 0.26	/	/
UPDRS-III	28.91 ± 4.52	/	/

*N, numbers; M, male; F, female; UPDRS-III, Unified Parkinson’s Disease Rating Scale III and Data (mean ± SD). ^a^Pearson chi-square test. ^b^Independent-samples *T* test*.

### Comparison of Concentrations of Serum HO-1, ppm Value of QSM, and Level of HGB in PD Cases and Controls

**Figure 1:**
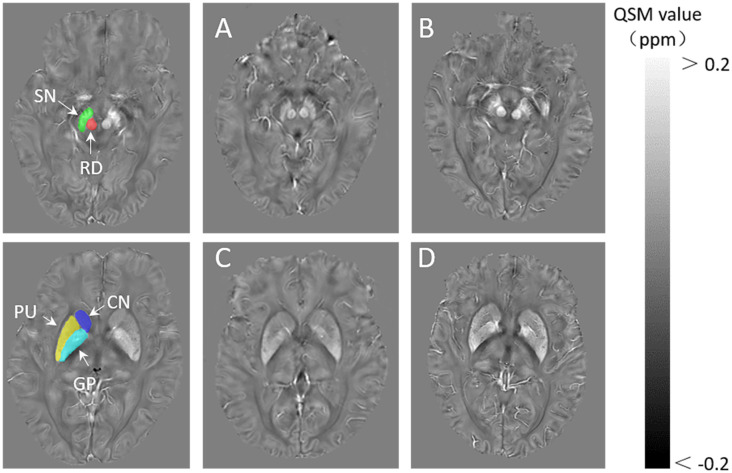
The definition of regions of interest (ROIs).The bilateral substantia nigra (SN), globus pallidus (GP), red nucleus (RN), the head of the caudate nucleus (CN), and putamen (PUT) are included. Representative images of quantitative susceptibility mapping (QSM): a 60-year-old healthy man **(A,C)**, a 65-year-old woman with PD **(B,D)**.

The mean levels of serum HO-1 were higher in the PD group than in the control group (*p* < 0.01). The PD group exhibited significant elevation in the mean QSM value of bilateral substantia nigra (*p* < 0.01), red nucleus (RN; *p* < 0.01), and putamen (*p* < 0.01) as compared to the control group. There was no significant difference in the mean QSM value of a bilateral head of caudate nucleus (CN), globus pallidus (GP) between the PD and the control groups, although the value for the former was higher than the latter. The mean levels of HGB were significantly lower in the PD group than in the controls (*p* = 0.027), even after controlling the confusing effect of gender (*p* = 0.044; Figure 1, Table 2).

**Table 2:** Level of heme oxygenase-1 (HO-1), hemoglobin (HGB), and regional QSM values (*10^−2^) in patients with PD and CG.

	PD	CG	*p*
*N*	32	26	/
HO-1(pg/ml)	85.685 ± 3.868	45.739 ± 3.686	0.000**
HGB (g/l)	127.500 ± 2.673	135.192 ± 1.827	0.044*
QSM value of ROIs			
SN	8.181 ± 1.548	6.623 ± 1.061	0.000**
RN	7.530 ± 2.003	5.657 ± 1.590	0.002**
CN	2.627 ± 0.848	2.410 ± 0.642	0.351
GP	7.700 ± 1.600	7.002 ± 1.071	0.104
PUT	3.568 ± 1.205	2.524 ± 0.811	0.002**

QSM, quantitative susceptibility mapping; SN, substantia nigra; RN, red nucleus; CN, the head of caudate nucleus; GP, globus pallidus; PUT, putamen. *PD vs. CG p < 0.05; **PD vs. CG p < 0.01.

### Correlation Between Serum HO-1 Levels and QSM Value, Level of HGB in Patients With PD

**Figure 2:**
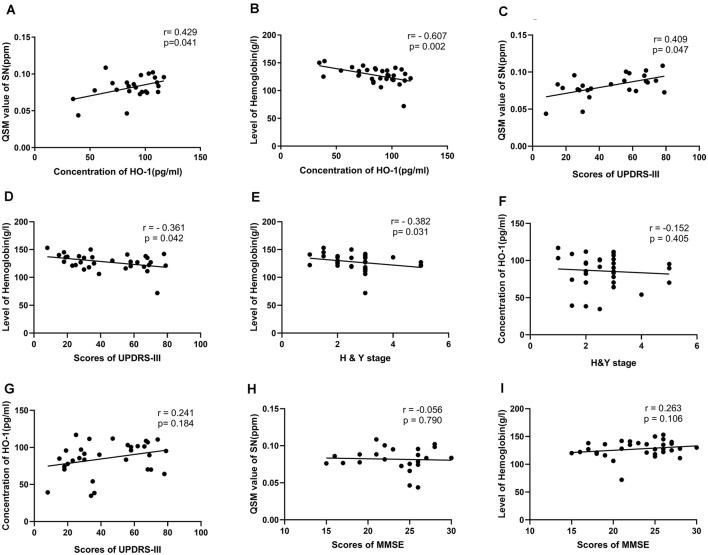
Correlations between the level of heme oxygenase-1 (HO-1) and the QSM values or hemoglobin (HGB), between HO-1 levels, QSM value, level of HGB and scores of clinical scales in PD patients. Levels of HO-1 correlated positively with mean QSM values of bilateral SN, but correlated negatively with levels of HGB **(A,B)**. Mean QSM values of bilateral SN correlated positively with scores of UPDRS-III. Levels of HGB correlated negatively with scores of UPDRS-III and H & Y stage. There were no significant correlations between serum HO-1 levels and scores of clinical scales, between mean QSM values of bilateral SN, levels of HGB and scores of MMSE **(C–I)**.

To explore the specific relationships between the elevation in serum HO-1 levels and increased QSM value as well as lower HGB of PD patients, a correlational analysis was conducted. The results revealed a statistically positive correlation between serum HO-1 levels and mean QSM values of bilateral substantial nigra (*r* = 0.429, *p* = 0.041), whereas there was no significant correlation between serum HO-1 levels and mean QSM values of other ROIs. The results also showed a significant, inverse correlation between serum HO-1 levels and mean levels of HGB in PD patients (*r* = −0.607, *p* = 0.002; Figures 2A,B). All *p*-values were adjusted for age.

### Correlation Between QSM Values of SN, Levels of HGB, Serum HO-1 Levels, and Scores of Clinical Scales in Patients With PD

To check whether the three factors (HO-1, HGB, and brain iron) correlate with worsening of PD or cognitive deficits, a correlational analysis was performed. The results revealed a statistically positive correlation between mean QSM values of bilateral substantial nigra and scores of UPDRS-III (*r* = 0.409, *p* = 0.047). The results also showed a significant, inverse correlation between levels of HGB and scores of UPDRS-III (*r* = −0.361, *p* = 0.042), H & Y stage (*r* = −0.382, *p* = 0.031). whereas there was no significant correlation between serum HO-1 levels and scores of clinical scales, between mean QSM values of bilateral SN, levels of HGB, and scores of MMSE (Figures 2C-I).

### Comparison of HO-1, HGB, and Value of QSM in Mice

**Figure 3:**
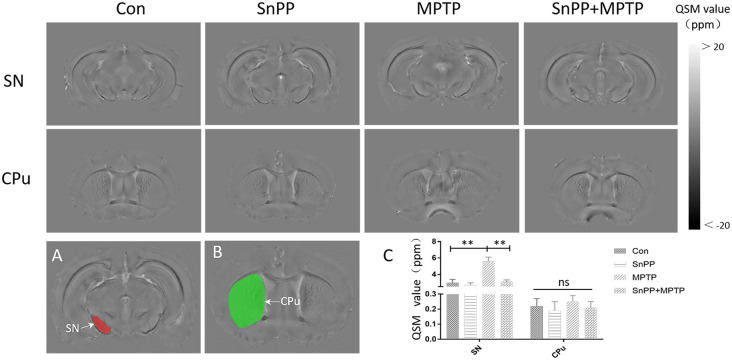
Representative images of QSM in different mice groups and the definition of ROIs.The ROIs included the substantia nigra **(A)** and striatum **(B)**. MPTP increased iron deposition in the SN in mice, while the HO-1 inhibitor (SnPP) inhibited iron deposition in the SN. However, no significant elevation in the QSM values of bilateral striatum was observed in all mice **(C)**. **p < 0.01; ns, not statistically significant.

The results revealed changes in HO-1, HGB, and the value of QSM in MPTP-treated mice. To verify the role of HO-1 in iron deposition and HGB reduction, HO-1 inhibitor (SnPP) was used to alter the function of HO-1. It was found that the MPTP mice exhibited significant elevation in serum HO-1 and mean QSM value of bilateral substantia nigra compared with the control mice. Further, SnPP significantly reduced the mean QSM value of bilateral substantia nigra induced by MPTP, though it could not bring the mean QSM value of bilateral substantia nigra down to the level of control mice. However, there was no difference in HGB and mean QSM value of striatum between the MPTP group and the control group. The results indicate increased HO-1 in the serum and reveal the role of HO-1 in iron deposition in the substantia nigra (Figure 3, Table 3).

**Table 3:** The comparison of Level of HO-1, HGB and regional QSM values in different groups of mice.

	Con	SnPP	MPTP	SnPP+MPTP	*P*_1_	*P*_2_
*N*	5	5	5	5
HO-1 (pg/ml)	41.15 ± 8.64	36.48 ± 3.25	94.15 ± 6.55	72.06 ± 3.99	<0.001	<0.001
HGB (g/l)	148 ± 7.7	143.8 ± 6.69	139 ± 8.65	141.80 ± 11.8	0.16	0.7
QSM value (ppm)						
SN	2.97 ± 0.14	2.73 ± 0.28	5.58 ± 0.52	3.1 ± 0.24	<0.001	<0.001
CPU	0.21 ± 0.04	0.19 ± 0.06	0.25 ± 0.04	0.23 ± 0.05	0.22	0.51

*P-values are presented as follows: *p*_1_: MPTP group vs. Con group; *p*_2_: MPTP group vs. MPTP + SnPP group*.

## Discussion

The current study suggests that the increase of iron deposition in the nervous system and the decrease of HGB may have common pathological factors. Further analysis showed that the abnormally high expression of HO-1 may be the foundation and bridge of the reported changes.

Studies have shown that iron deposition in the SN has a pivotal role in the necrosis of dopaminergic neurons (Dexter et al., 1987), the aggregation of α-synuclein (Ostrerova-Golts et al., 2000; Barnham et al., 2004), and promotes the progression of the disease. Iron deposition is not only an important pathological marker of Parkinson’s disease (PD) but also an important driving factor for its progression. Currently, QSM is widely used in the examination and diagnosis of diseases characterized by iron deposition.

In addition to brain iron deposition, recent studies revealed that patients with anemia were more susceptible to PD. In other words, lower HGB may be a risk factor for PD (Savica et al., 2009). Our primary (Deng et al., 2017) and current results suggested that HGB levels were lower in PD patients compared to the controls. Some studies have shown that a decrease in HGB precedes the onset of motor symptoms. Therefore, this phenomenon could not be solely explained by nutritional disorders.

Nevertheless, the specific mechanisms of iron deposition and lower HGB level in PD are unclear. Iron deposition is certainly a complex and comprehensive process, but it must have a major fundamental process and cause. Increased iron uptake or failure of iron export might be contributing factors (He et al., 2015). The current study shows that PD is a disease involving multiple systems, anemia or lower HGB may indicate intrinsic pathological changes of PD. Also, since iron is an important metal ion bound to HGB, there is a need to consider the two pathological features together to establish the mutual underlying mechanism between them.

As an antioxidant, HO-1 has attracted more attention in the research of Parkinson’s disease. In addition to the over-expression of HO-1 in the brain of PD patients (Schipper et al., 2019), some previous studies indicated possible overexpression of HO-1 in peripheral blood and saliva of PD patients, compared to the controls (Mateo et al., 2010; Song et al., 2018). Besides, Genetic studies also support the association of HMOX gene variations with the risk of PD, for instance, some HMOX1 gene variants increased the risk of some forms of PD (Ayuso et al., 2014). However, a previous study did not report a significant association between genetic markers in the HO-1 gene with increased susceptibility to PD (Funke et al., 2009). Based on the relevance of HO-1 in iron metabolism, investigations were carried out to determine the relationship between the level of HO-1 with brain iron deposition as well as the lower HGB level of PD patients. Consistent with previous findings (Mateo et al., 2010), the current study showed that serum HO-1 levels are increased in PD patients, compared with controls. More importantly, the elevation of HO-1 level positively correlated with the iron deposition of SN, and inversely with the HGB level in PD patients. The animal model study confirmed that HO-1 inhibitor could significantly reduce the iron deposition in SN. Therefore, it was suggested that the high expression of HO-1 in SN may be associated with iron deposition. Since the HO-1 is the key enzyme for heme catabolism, while heme is the main structure of HGB, the over-expression of HO-1 in peripheral blood and central nervous system in PD patients may increase the catabolism of heme, thus decrease the concentration of HGB. Though the current study did not test the heme decomposition products, a previous study (Macías-García et al., 2019) has concluded that overexpression of HO-1 could lead to higher peripheral bilirubin levels in PD, a major heme decomposition product. Also, with the catabolism of heme, the iron released from heme may aggravate the iron deposition in the brain. A previous study indicated that inhibition of heme oxygenase could ameliorate anemia and reduce iron overload in a β-thalassemia mouse model (Garcia-Santos et al., 2018).

Interestingly, the elevation of HO-1 positively correlates with the iron deposition of SN, but not in other regions. Some reasons could explain this interesting observation. First, although Parkinson’s disease affects multiple nerve sites, dopaminergic neurons that are concentrated in the substantia nigra are still the main lesion. Both nigra neurons and astrocytes of PD brain specimens exhibited intense HO-1 immunostaining (Schipper et al., 1998), which suggested continuous oxidative stress. Over-expression of HO-1 could derange iron homeostasis and aggravate iron deposition in SN. Second, the over-expression of HO-1 and increased iron deposition of SN could be connected to chronic inflammation. Chronic neuroinflammation involves glial activation and peroxidation, and HO-1, as an important antioxidant factor, would play a role in this critical course. A previous study (Urrutia et al., 2014) indicated that a vicious cycle of exacerbated oxidative stress and increased iron accumulation could be promoted by inflammatory processes. On one hand, the gene expression of iron regulatory factors would change and iron deposition would exacerbate under inflammatory conditions (Recalcati et al., 2012). On the other hand, the increased iron deposition could generate secondary inflammatory response (Liu et al., 2017) and oxidative stress.

Also, the animal experiments revealed that although the serum HO-1 of PD model mice increased, the HGB did not decrease significantly. Two scenarios were considered as possible explanations for this observation. First, mice could have a great ability of compensation; second, the lesions involved in MPTP-induced PD model were different from PD patients, because PD patients’ lesion is more extensive, including gastrointestinal tract/heart/spinal cord and other parts (Hawkes et al., 2007). Therefore, the source of HO-1 in PD patients may be more extensive and stable.

We found that iron deposition and levels of HGB were associated with motor symptoms, whereas there was no correlation between levels of HO-1 and disease severity. We speculate that one possible reason is the dynamic change of level of HO-1, higher in the early stage and lower in the late stage of the disease (Song et al., 2018). With the progression of the disease, the number of nigra neurons is greatly reduced, and the neuroinflammation may gradually weaken. Therefore, the expression of HO-1 would not sustain a high level.

It is noteworthy that this study had some limitations. First, the sample size was relatively small. Future research should be based on a larger sample size to verify the results. Second, a follow-up study was not conducted to evaluate dynamic changes of HO-1 and its association with brain iron deposition and HGB. Third, the gender ratio of this study might induce a bias, even after controlling the confusing effect. Last but not least, heme decomposition products were not analyzed in the current study. This calls for more comprehensive studies in the future. Despite these deficiencies, this study provides the first report on the association between peripheral HO-1 and HGB, brain iron deposition in PD patients.

## Conclusion

In summary, the current study suggests that brain iron deposition is increased and HGB is significantly reduced in patients with PD, due to over-expression of HO-1.

## Data Availability Statement

The raw data supporting the conclusions of this article will be made available by the authors, without undue reservation.

## Ethics Statement

The studies involving human participants were reviewed and approved by Ethics Committee of Nanfang Hospital. The patients/participants provided their written informed consent to participate in this study. The animal study was reviewed and approved by Ethics Committee of Nanfang Hospital.

## Author Contributions

JX: conceptualization, data curation, formal analysis, methodology, investigation, visualization, and writing—original draft. CX: data curation, formal analysis, methodology, investigation, visualization, and writing—original draft. WS: data curation, formal analysis, and investigation. XC: formal analysis, methodology, and supervision. MP: data curation, formal analysis, and methodology. QW: conceptualization, funding acquisition, and writing—review and editing. YF: methodology, supervision, validation, and writing—review and editing. YX: conceptualization, funding acquisition, methodology, resources, supervision, and writing—review and editing. All authors contributed to the article, read and approved the submitted version.

## Conflict of Interest

The authors declare that the research was conducted in the absence of any commercial or financial relationships that could be construed as a potential conflict of interest.
